# Challenges and Experiences of Managed Aquifer Recharge in the Mexico City Metropolitan Area

**DOI:** 10.1111/gwat.13237

**Published:** 2022-08-24

**Authors:** Adriana Palma Nava, Timothy K. Parker, Rafael B. Carmona Paredes

**Affiliations:** ^1^ Instituto de Ingeniería, UNAM, Av. Universidad No. 3000 Col. Universidad Nacional Autónoma de México, Coyoacán Ciudad de México C.P. 04510 Mexico; ^2^ Ramboll USA 2200 Powell Street Emeryville CA 94608

## Abstract

The Mexico City Metropolitan Area (MCMA) is a significant and important urban center in North America, covering an area of approximately 9500 km^2^ with a population of almost 23 million, yet the water supply remains unsustainable. The total water demand in the MCMA is 84 m^3^/s and is provided by groundwater (63% or 53 m^3^/s), imported water (27% or 23 m^3^/s) and recycled water (10% or 8 m^3^/s). The natural recharge of the MCMA aquifer is approximately 23 m^3^/s, indicating an overexploitation of groundwater resources of approximately 25 m^3^/s (800 Mm^3^ annually), a reasonable future goal for recharge in the MCMA. Hydrologic analysis indicates two main opportunities currently to increase water supply in the MCMA: indirect water reuse with recycled water and managed aquifer recharge (MAR) with storm water. An inventory of MAR project case studies in the MCMA summarizes methods for recharge, water sources, geographical distribution, and the main results obtained in each project for the last 80 years. The inventory consists of 21 MCMA area MAR case studies including (1) conceptual, (2) design level, and (3) pilot‐ to full‐scale facilities, only some of which have operated for relatively short periods of time, with one remaining MAR project currently operational. The review found that beyond the technical and economic issues that MAR project design normally address, the existing regulatory framework and the continuous change in water district chairs in charge of the operation and supply of water are significant barriers to increasing MAR in the MCMA.

## Abstract 2

*Article impact statement*: It provide an state of art on MAR en Mexico City Metroplitan area and could be a reference to develop an inventory.

## Introduction

The Mexico City Metropolitan Area (MCMA) contains the nation's capital, Mexico City, one of the most important areas in the country in terms of history, national economy and culture, with a current population of circa 23 million, and constantly growing. The water supply of the extended metropolitan area (urban, agriculture, and industry) depends on imported water from the Lerma and Cutzamala systems (25%), rivers and springs (3%) and groundwater resources (72%). A large portion of the MCMA is covered by urban hardscape reducing recharge potential dramatically, and most of the associated runoff and waste water is conveyed out of basin for flood control, treatment and reuse, with a rather small in basin reuse of recycled water (Palma‐Nava et al. 2022).

Groundwater levels have been chronically declining since the 1960s with associated increasing costs of pumping and some wells going dry. Thus, the security of the city's economy, social stability, and water supply strongly depends on a diminishing, unsustainable supply of groundwater. Increasing water demands have resulted in overexploitation of the groundwater resources in the area over several decades.

Managed aquifer recharge, also called groundwater replenishment, water banking and artificial recharge, is the purposeful recharge of water to aquifers for subsequent recovery or environmental benefit. MAR methods include riverbank filtration, stream bed weirs, infiltration ponds and injection wells, and uses water sources including appropriately treated urban stormwater and treated waste water to increase groundwater storage, protect and improve water quality, and secure drought and emergency supplies. A growing scientific information base supports rapidly increasing MAR as a vital management tool in the sustainable use of the world's water resources.

**Figure 1:**
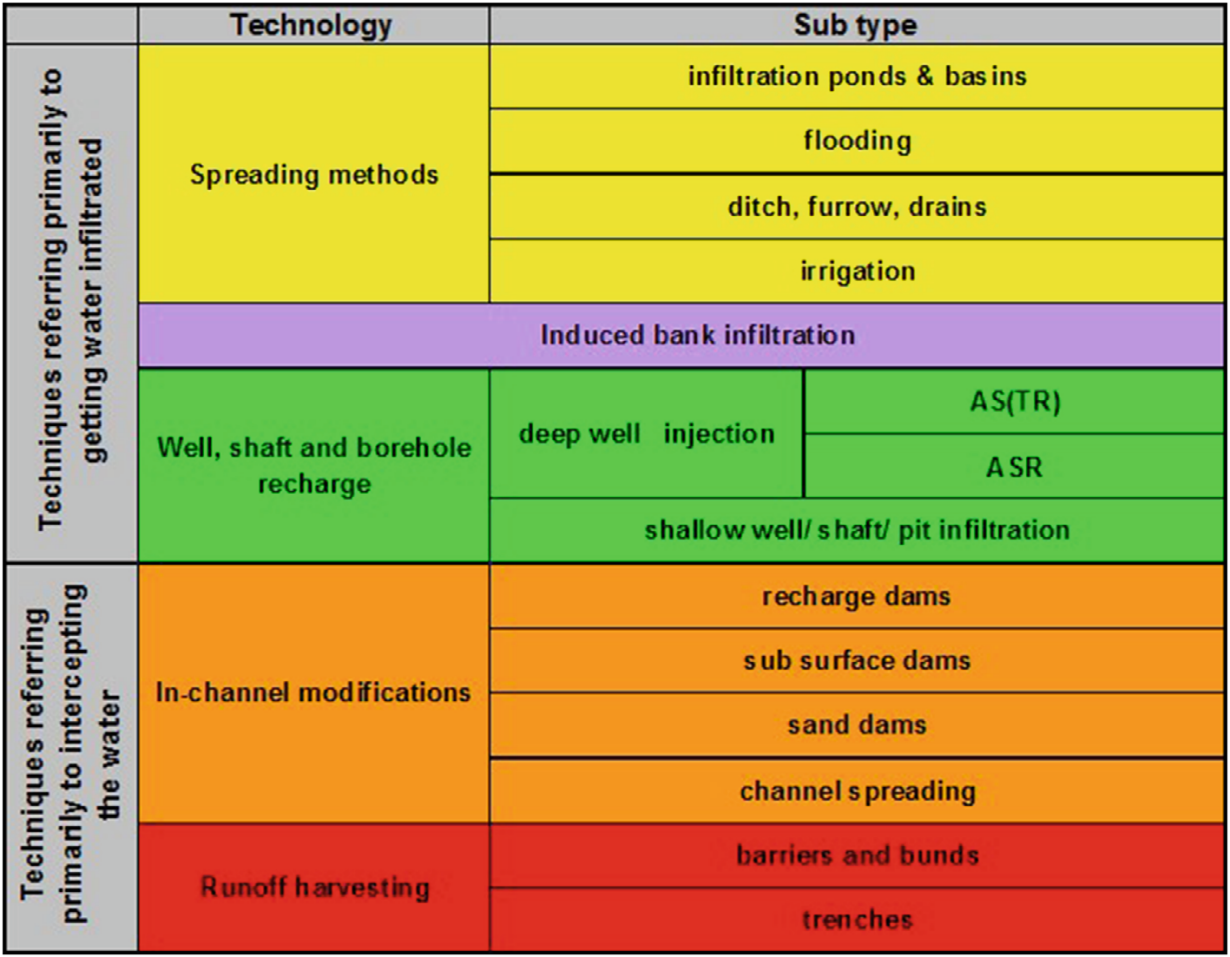
Classification of MAR technology and applications (subtype) (IGRAC 2014).

The technologies to implement managed recharge are multiple and varied. However, five principal technologies with 14 subtypes (applications) are identified based on whether the technology focuses on the method of aquifer recharge or is focused on water interception (capture) for subsequent surface infiltration (Figure 1—IGRAC 2014).

There have been numerous efforts and research to study, pilot test and implement MAR projects in the MCMA, based on the recognition that MAR is a proven technology, and sufficient surface water, storm water and treated waste water exist to significantly offset groundwater demand with recharge. This article provides an inventory of MCMA MAR projects, a summary of some of the challenges and lessons learned, and some suggested next steps for increasing MAR in the MCMA in the future.

## Aquifer Conditions in Mexico

**Figure 2:**
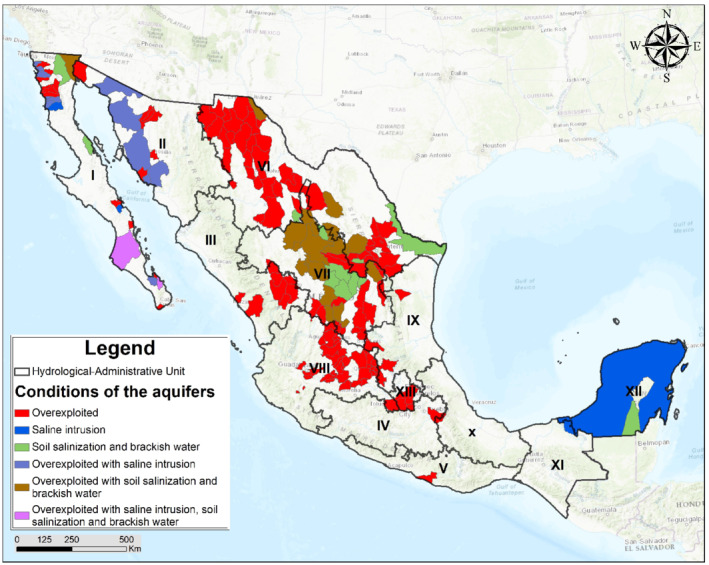
Status of aquifer conditions in Mexico (CONAGUA 2018).

Although MAR has not been used extensively in Mexico, there is a history of the application and management of these practices to try to address the increasing overexploitation of the country's aquifers. Mexico has defined 653 administrative aquifers (Figure 2) that meet the majority of industrial demands and about 70% of the water required by cities to supply approximately 60 million people. The number of designated aquifers in Mexico has grown significantly over time: 32 aquifers designated overexploited in 1975 and 123 in 2017 (CONAGUA 2018). Generally, Mexico's groundwater quality is adequate, with 80% of the aquifers designated good quality water. Overexploitation is taking its toll on quality though, with 18 aquifers identified with sea water intrusion, and 32 with overlying soils salinization and associated brackish groundwater (CONAGUA 2018). The MCMA overlies the Mexico Basin, a designated overexploited aquifer, located largely within Hydrological‐Administrative Unit XIII (Figure 2).

## MCMA Setting

**Figure 3:**
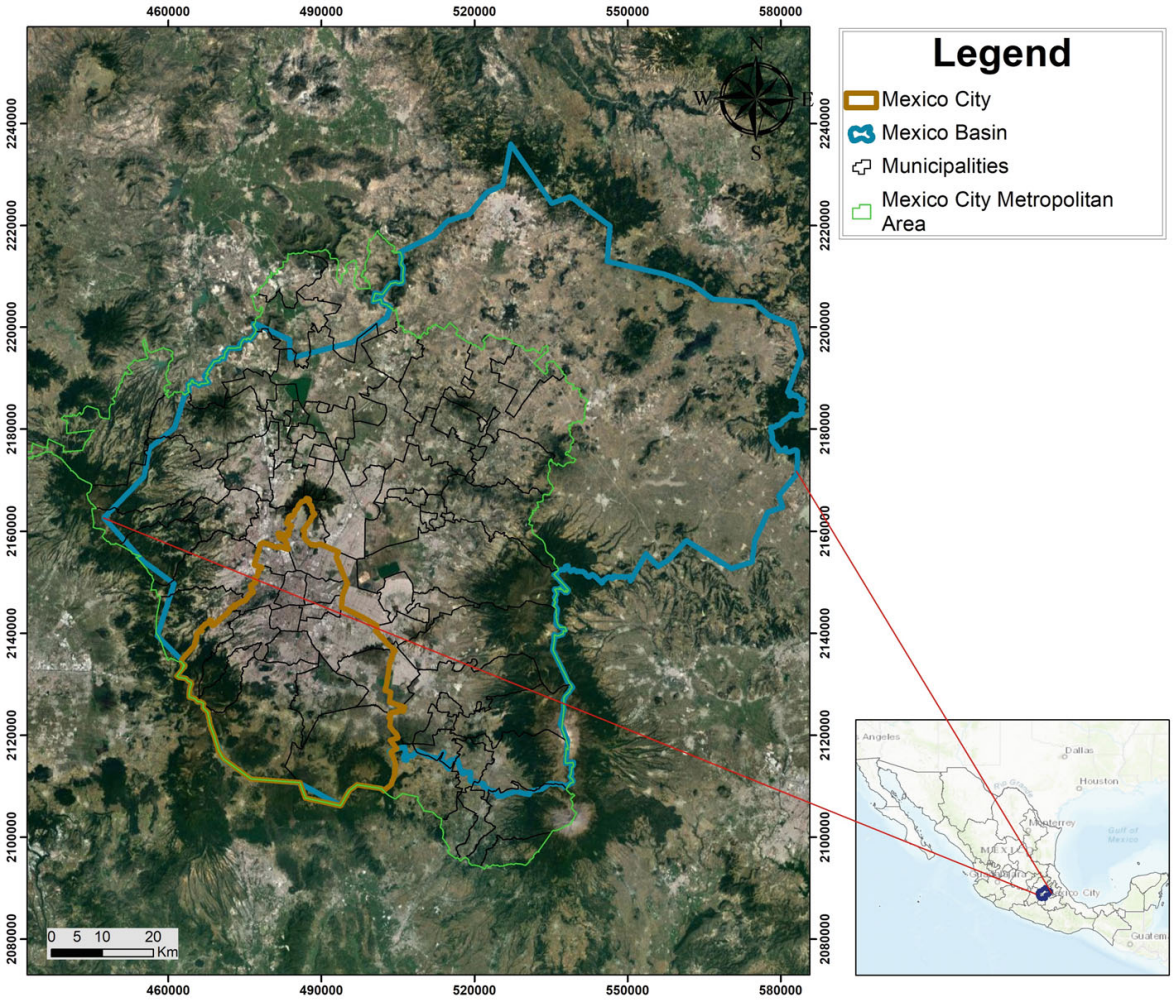
Extent of the MCMA.

The MCMA is located dominantly within the Mexico Basin, a closed basin with an average elevation of approximately 2200 m above sea level, and is surrounded by mountains and volcanoes of the Trans‐Mexican Volcanic Belt that reach over 5000 m elevation (Figure 3). The closed basin setting created a series of lakes that prior to development, covered the basin floor, and resulted in the formation of thick lacustrine deposits that the MCMA now overlies. Drainage was engineered in the 1600s to manage flooding by conveying the storm water largely out of the basin, and eventually draining virtually all of the lakes. The area receives approximately 820 mm of annual rainfall from May through October much of which is still conveyed out of the basin (50 m^3^/s), while groundwater resources continue to be overexploited and depleted, a classic tragedy of the commons. The current estimated amount of groundwater extracted in the MCMA is 53 m^3^/s or 1670 Mm^3^/year, resulting in an ongoing overexploitation rate of 25 m^3^/s or nearly 800 Mm^3^/year (Palma‐Nava et al. 2022). Undesirable results of groundwater depletion that are evident include chronic, cumulative groundwater level declines averaging 70 m in the MCMA, wells going dry and increasing pumping costs, land subsidence as much as 9 m from groundwater extraction, associated loss of groundwater storage volume and capacity (from aquifer compaction/subsidence), and water quality degradation. Mexico City is subsiding at an average rate of 30 cm/year (Santoyo et al. 2005) with associated significant economic and technical challenges, including issues with runoff, waste water management and flooding during wet, humid summer months. In view of the annual water available from precipitation, increasing recharge through MAR projects is a key water management tool to assist in reducing overexploitation and the associated negative impacts on Mexico's aquifers.

Considering the status of the decline of Mexico's aquifers, a fundamental first step to facilitate increasing MAR in this highly urbanized region would be to provide appropriate incentives for local users to implement MAR, and institute specialized training of the groundwater and water professional workforce. MAR needs to be employed much more widely on the supply side in order to replenish depleted aquifer systems and sustain groundwater resources in the future (Palma‐Nava et al. 2018). Additionally, increases in conservation and water use efficiency, and reduction of allocations as necessary also need to occur on the demand side (Gonzalez‐Villarreal et al. 2014).

## Inventory of MAR in MCMA

The results of an inventory of MCMA MAR case studies, including the phase and status of each MAR project, potential positive and negative impacts on associated aquifers, and the main benefits and challenges faced by MAR, are described in the following sections. The inventory has been compiled into the MCMA MAR case study inventory database, which aims to provide guidance for the planning and implementation of new MAR projects in this area. Results show that MAR projects have been distributed across the MCMA, with a majority concentrated in Mexico City.

### Methodology

The inventory of MAR project case studies was compiled from readily available scientific papers, conference proceedings, internal reports, and published academic theses dating back to 1943. A total of 23 MAR project case studies were identified and categorized following the IGRAC classification (Figure 1) for the MAR technology and sub‐type (application). The authors selected the IGRAC classification system to make the resulting information compatible with the IGRAC's Global Groundwater Information System, to facilitate access and promote international sharing of information and knowledge on MAR (Stefan and Ansems 2018). The MAR project case study inventory also includes the year, implementing institution, geographic area site location, source water and annual infiltration volume.

### Inventory Case Study Results

**Figure 4:**
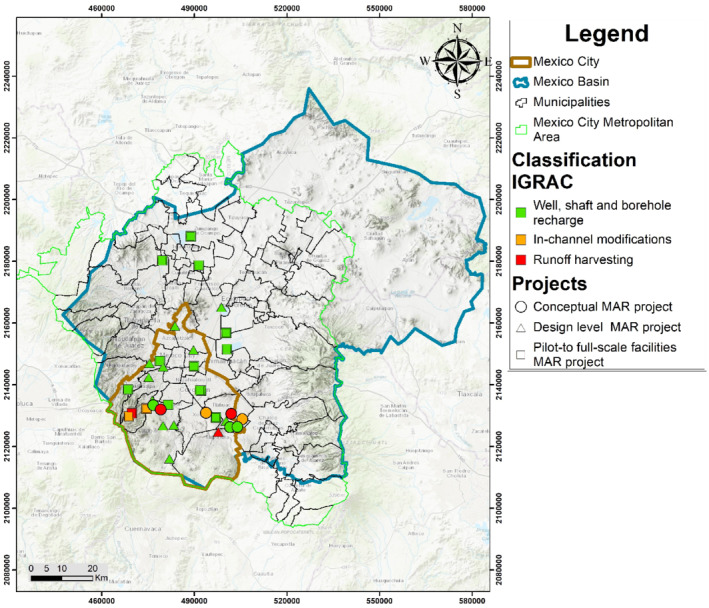
MAR projects localization into MCMA.

The compiled data from the inventory was classified in three main categories: (1) conceptual projects, (2) design‐level projects, and (3) implemented projects, summarized in Tables 1, 2, 3, respectively. Conceptual projects were developed at a screening level with the MAR method and type of technology, source water and target annual recharge volume, and some limited hydrogeological analysis. Design‐level projects were evaluated in much more detail and may already be at or close to shovel ready for implementation. Most of the executed projects were operated for only a few years (column one of Tables 1, 2, 3), with only one MAR project that remains operating today in the MCMA. Figure 4 shows the distribution of all the MAR inventory projects in the MCMA. All the projects are included in the references.

**Table 1:** Conceptual MAR Projects

Year	Institution	Project	Site	Application	Type of Water Used for Recharge	Recharge Volume (Mm^3^)
1964	Hydrological Commission of the Valley of Mexico Basin	First estimates of infiltrating the flow of the rivers of the Valley of Mexico basin	Xochimilco, Churubusco, Mexico City, Cuautitlan, Pachuca, Teotihuacan, Texcoco, Chalco, Apan	Controlled flooding	River water	284.51
2010	Mexico City Water System	Artificial recharge of groundwater with rain water and treated waste water	Xochimilco‐Tlahuac corridor	Infiltration lagoons	Treated waste water	110.0
2010	Mexico City Water System	Artificial recharge of groundwater with rain water and treated waste water	600 potential sites (until 2010, 135 were studied) (South of CDMX)	drainage well	Rainfall water	NA
2010	Mexico City Water System	Artificial recharge of groundwater with rain water and treated waste water	Tlalpan, Cuajimalpa, Milpa Alta, Xochimilco, Magdalena Contreras and Álvaro Obregón	Subsurface dams	River water	NA
2010	National Water Commission	Water Sustainability Program for the Valley of Mexico Basin. Artificial recharge of groundwater and water reuse programs	Sanitation Unit El Caracol	Injection well	Treated waste water	30

**Table 2:** Design Level MAR Projects

Year	Institution	Project	Site	Application	Type of Water Used for Recharge	Recharge Volume (Mm^3^)
1995	Department of Federal District. General Direction of Construction and Hydraulic Operation	Artificial recharge of groundwater with treated waste water Master plan.	RWTP Acueducto de Guadalupe, San Juan de Aragón, Bosques de las Lomas, Campo Militar, San Juan Ixtayopan, Abasolo, H. Colegio Militar, Parres, San Miguel Xicalco, Santa Fe	Injection well	Treated waste water	18.50
1997	Department of Federal District General Direction of Construction and Hydraulic Operation	Technical‐economic feasibility study for the recharge of the ZMCM aquifer	Chichinautzin Mountain Range (Xochimilco y Tláhuac) Phase I and II	Controlled flooding	River water	310
2011	National Water Commission	Artificial recharge of groundwater at experimental module in Texcoco Lake	RWTP Lodos activados	Injection well	Treated waste water	30
2012	National Water Commission	Instantaneous and short‐term recharge tests (active and passive), El Caracol artificial recharge pilot project	Lake Texcoco Federal Zone, Ecatepec	Injection well	Treated waste water	NA

**Table 3:** Implemented MAR Projects

Year	Institution	Project	Site	Application	Type of Water Used for Recharge	Recharge volume (Mm^3^)
1943–1960	Hydrological Commission of the Valley of Mexico Basin	First river diversions	River diversion from Magdalena to San Angel basalts	Controlled flooding	River water	73.5
1953–1954			River diversion from Eslava to Xitle basalts	Controlled flooding	River water	NA
1953–1958	Hydrological Commission of the Valley of Mexico Basin	First experiences using absorption wells	San Fernando well	Drainage well	Rainfall water	0.30
1955–1960			Private offices in Mexico city	Drainage well	Rainfall water	4.50
1955–1975			Mixcoac	Drainage well	Rainfall water	34.70
1977	Moreno Pecero (1977)	Differential land subsidence control	Palacio Nacional	Injection well	Potable water	—
1987	National Water Commission Texcoco Lake Commission	First experimental aquifer recharge module	Experimental injection wells with treated waste water	Injection well	Treated waste water	NA
1989–1992	Hydrological Commission of the Valley of Mexico Basin	General artificial recharge program for Mexico City	RWTP San Luis Tlaxialtemalco, Ciudad Deportiva, Cerro de La Estrella	Injection well	Treated waste water	10.90
1991	Figueroa (1991)	Differential land subsidence control	Centro Cultural Universitario Tlatelolco	Injection well	Potable water	—
1991	Pliego y Vargas (1991)	Differential land subsidence control	Conjunto Hidalgo	Injection well	Potable water	—
2009	Mexican Institute of Water Technology	Recharge using rain water in the Magdalena river basin, Mexico City	Magdalena river	Subsurface dams	River water	0.5
2014	Mexico City Water System	Artificial recharge of groundwater in the Cerro de La Estrella area with treated waste water	Cerro de la Estrella	Injection well	Treated waste water	NA
2017	National Water Commission	Artificial recharge in Lake Texcoco (NAICM)	Lake Texcoco, NAICM area	Drainage well	Rainfall water	NA
2017	SUEZ	Project for reuse and artificial recharge of groundwater in Chapultepec, Mexico City	RWTP Chapultepec	Injection well	Treated waste water	NA

Abbreviation: NA = not available.

### MAR Objective

In general, each of the projects is characterized by presenting more than one objective. The IGRAC classification (IGRAC 2014) identifies six main objectives presented by MAR projects: (a) maximization of groundwater storage, (b) water quality management, (c) physical management of aquifers, (d) management of water distribution systems, (e) ecological benefits, and (f) other benefits, which for this area is land subsidence management.

Except for the conceptual projects, the main objective in more than 79% of the case studies is to stabilize and restore groundwater levels and halt subsidence. Other benefits included flood mitigation in 15% of the case studies, and 5% included maximization of groundwater storage. One project also includes differential land subsidence control as an objective.

### MAR Project Operating Status

As mentioned previously, of the 23 MAR case studies in the MCMA MAR inventory, five are conceptual and four design level, and 14 have been operated although mainly for a relatively short period of time. Currently there is only one MAR project operating, and that is a pilot level injection project using recycled water from the Chapultepec Wastewater Treatment Plant (Chapultepec WTP).

The Chapultepec WTP facility, located in Molinos del Rey in Chapultepec of the MCMA, was established with the objective to increase water reuse, reduce water scarcity, preserve the forest and lakes of Chapultepec, and improve the water quality of the artificial lakes in Chapultepec. The facility treats approximately 170 cubic liters per second domestic waste water using pretreatment, biological reactor for sludge removal, biological membrane technology, and ultraviolet disinfection for nonpotable uses, including water for recreational activities by supplementing the surface waters of the lakes of Chapultepec, and for nearby park and forest irrigation. Water from the lakes of Chapultepec is diverted and further treated using ultrafiltration, reverse‐osmosis and ultraviolet disinfection prior to injection through wells to replenish the Mexico

Basin aquifer. Originally funded by CONAGUA, as of the time of the preparation of this paper, the management of the plant and recharge project are in transition, and the current project status is unavailable (World Bank 2020).

A number of factors have been identified that led to the majority of the implemented projects only being operated for a short time including improper facilities design. Inadequate or incorrect operation, maintenance and monitoring; unexpectedly high costs associated with the MAR operations, maintenance and monitoring programs; and inadequate data on source water and receiving water quality and the potential negative impacts on mixing. Unfortunately, there is little information available, or data collected on the operating parameters for most of the implemented MAR projects in the MCMA. Considering that all but one project were unsuccessful and ceased underscores the need for more basic initial characterization studies, iterative building and upscaling of MAR projects, technology and knowledge transfer, and addressing regulatory, legal and political constraints to increase potential success on MAR in the MCMA.

### MAR Project Technology and Application

The technology and application are summarized to provide an understanding of the case studies in the inventory. Sixteen case studies include well, shaft, borehole technologies as described by IGRAC (2014) and of these, 11 are deep well applications and the remaining five are shallow wells (drainage, vadose zone or dry well). The injection well projects use recycled water and all five shallow drainage wells recharge rainfall runoff.

Five of the case study projects involve surface infiltration by spreading. Of these, one involves an infiltration lagoon recharging recycled water and the other four are controlled flooding of stream flows. The two remaining case study projects are both in‐channel modifications that use subsurface dams to increase recharge of stream flows.

### MAR Source Water

A significant consideration for a MAR project is the availability and reliability of source water for recharge. Other important aspects to consider for the source water are the water quality and potential variability of quality, conveyance and the location of the intake and recharge points, source water rights, and any associated costs and fees. In the MCMA MAR case studies, four main source waters have been identified: perennial stream flows (surface water), rainfall runoff, potable water and recycled water. The case study source waters are approximately 60% with recycled water, 25% stream flows, while the remaining 15% are rainfall runoff.

### Recharge Volume

Tables 2 and 3 provide the volume of the annual volume and the type of water proposed or used for recharge. The total volume of conceptual, design level and implemented MAR projects sum to over 900 Mm^3^, or more than the current overdraft of the MCMA area, if all the conceptual, design level and executed case study projects were implemented and operational. The implemented projects total nearly 125 Mm^3^/year or roughly 16% of the overexploitation of the MCMA.

Notable projects that were conceived in the MCMA include surface spreading by controlled flooding of diverted stream flows proposed in the early 1960s by the Hydrological Commission of the Mexico Valley Basin. This included diversion and spreading facilities at eight locations across the region proposing approximately 285 Mm^3^/year recharge. Another notable project that is at design‐level proposed by the Department of the Federal District involved a similar approach of spreading with controlled flooding of stream flows at two locations amounting to 310 Mm^3^/year. These two projects together, if operational, would address nearly 75% of the MCMA overexploitation.

## MAR Regulatory and Legal Framework

Mexico is one of the few countries that have national regulations for the development and implementation of MAR projects. The official Mexican standards for MAR include NOM‐014‐CONAGUA‐2007 (DOF 2009a), and NOM‐015‐CONAGUA‐2007 (DOF 2009b) to protect aquifers when recharging with recycled water and rainfall runoff/stormwater, respectively. Both standards consider the unsaturated zone for natural soil treatment of recharged water that can be used with an appropriate combination of pretreatment compatible with the recharge method. The standards are silent on the rights to the recharged groundwater.

### Disincentives for Water Suppliers to Recharge

In Mexico, all water is federally owned, and the federal organization responsible for permitting and maintaining water in the country is CONAGUA (the National Water Commission). All water is controlled through assignment of concessions (water rights), and all water has a unit fee that is required by federal law to paid to CONAGUA by local water agencies. The local agencies that have responsibility for water supply, waste water, and flood control are the government institutions that have the authority to do MAR projects, but there are no incentives for implementation of MAR projects, in fact there are significant disincentives.

The existing legal framework has no provisions to provide the local agency that recharges groundwater the legal right to recover the recharged water. All recharged water is federal property, meaning that if a local agency invests in the capital to construct an MAR project, that local agency will not have the right to recover that water, and would first have to obtain the additional concession and also pay CONAGUA to recover the recharged groundwater. This unfortunately translates into a disincentive for all Mexican local water agencies to make any capital investments in MAR, because it is less costly and less effort to simply continue depletion of groundwater supplies.

Additionally, reclaimed water lacks definition in the law and how it can allocated, providing uncertainty in terms water rights to begin with. If a water agency recharges treated waste water, they lose their rights to the recharged water, providing a disincentive to recharge treated waste water (Cruz‐Ayala and Megdal 2020).

### Balancing Quality and Quantity

Another challenge that has been identified within the regulatory framework in Mexico which similarly occurs in other countries, is that there is not a way to balance the benefits of an increased or sustainable quantity of supply with some acceptable level of groundwater quality degradation for regulatory acceptance. Groundwater that receives recharge (source) water inevitably involves some increment of quality changes, either in a positive or negative way, due to the mixing of two chemically different waters. For example, recharging groundwater with higher total dissolved solids (TDS) source water will predictably increase the TDS level in the groundwater over time. If recharging can address water supply reliability for a given area, with an incremental but locally acceptable degradation of the groundwater quality, then there should be a way to balance the issue between change in quality and increased quantity of supply.

### Community Outreach and Public Involvement

Additional aspects that are lacking in the regulatory and legal framework are requirements for public involvement of local communities and stakeholders in devising and implementing projects. Community outreach and coordination with local stakeholders, including the federal, state and local governments, utility agencies, committees and councils, is key to building public trust, ensuring input and acceptance of projects.

In summary, development of incentives, including regulatory and legal (water rights), financial or other, to encourage local agencies to implement MAR projects is essential to the future of the MCMA. There are many examples of incentives for MAR projects, including giving the recharger the right to recover the water with no additional fee, grants and subsidies to help pay for capital investments, many of which have associated requirements such as public outreach and stakeholder involvement, data collection and reporting, green infrastructure, and making MAR projects multibenefit (e.g., recharge, recreational, and ecosystem services). Without changes to the regulatory and legal framework and incentivizing recharge, the MCMA is destined to continue on the path of further groundwater depletion and related land surface subsidence, with the associated economic impacts. The groundwater is finite and eventually will no longer support the MCMA needs.

## Discussion

The MCMA has limited experience with MAR projects, little success in long‐term project implementation and actually getting much water into the ground compared to the overexploitation. Based on the inventory and research conducted under this work, we find that the limited successes are due to a number of factors, including issues with planning, design, operation and maintenance, but more so, the regulatory, legal and political framework that presents barriers to MAR and disincentives for implementing MAR projects.

The largest technical challenge for MCMA MAR projects is clogging, the causes of clogging are well described (Martin 2013) and include mechanical effects of the device, the biological activity, and the chemical processes of the source water interaction with the receiving environment. Potential clogging impacts can be managed and engineered with appropriate MAR project planning, design, implementation, monitoring and maintenance.

Maintaining the MAR project design infiltration rate is critically important as it relates to meeting the MAR project objectives and being able to utilize all the source water when it is available. MAR facility maintenance is key to sustaining desired infiltration and injection rates, and involves cleaning, scouring and scraping infiltration surfaces, and backwashing and cycling injections systems. After a certain time and volume of water recharged, the devices may have been abandoned, as they could not regenerate their infiltration capacity with the original operating flow rates. This process of capacity degeneration frequently translates into the need to estimate the useful life of the facilities and carry out relevant economic studies to quantify their cost‐benefit ratio, and to improve design and maintenance practices.

The largest infiltration volume projects involve capturing and recharging perennial stream flow diversions as expected, as this is the largest source water volume available. The second largest source water volume identified is recycled water, followed by rainfall runoff. Considering that the average annual precipitation in the MCMA is approximately 800 mm, the MCMA area could focus more efforts on recharging stormwater instead of conveying it out of basin. Additionally, rainfall runoff could be captured through basin scale low impact development (LID) techniques and green infrastructure in both new developments and retrofits in existing developments, reducing the urban hardscapes and promoting the slow‐it, spread‐it, sink‐it methods in land use so that more of the runoff is recharged for later recovery. Notably, the total volume of conceptual, design level and implemented MAR projects total nearly 900 Mm^3^, which is greater than the current overdraft of the MCMA area.

It is worth once again noting the complete absence of economic incentives in Mexico to promote or subsidize the planning and execution of MAR projects. Capital costs for MAR projects are expensive and complex when considering the legal, institutional, and economic aspects, especially the uncertainty of recovering the recharged water under the existing water rights system and identifying the beneficiaries and who pays. Government incentives and subsidies have proven to be successful in increasing recharge elsewhere and should be implemented in Mexico to promote the increase in recharge in the MCMA focused on the areas with greatest groundwater level declines and associated subsidence.

## Summary of Lessons Learned and Next Steps

It is clear that the MCMA and other areas of Mexico have critical problems of overexploitation of groundwater resources causing depletion and associated subsidence and groundwater quality degradation. Without action to mitigate the causes, the MCMA is heading to a tipping point where water supply will not be able to meet demand and the aquifer system that the MCMA relies on for nearly two‐thirds of its supply will be damaged and not recoverable.

The most significant challenge to increasing recharge in Mexico is the regulatory and legal framework that presents a disincentive, because the recharged groundwater belongs to the feral government and not the recharger. This needs to change and instead become an incentive to increase MAR projects and soon. Federal government financial and other incentives tied to MAR project reporting, public involvement, monitoring, data collection and reporting, increasing green infrastructure, and implementing multibenefit MAR projects.

Other challenges include technical aspects such as clogging, inadequacies in MAR design, operation and maintenance, and cost‐benefit planning. Technical and cost‐benefit aspects are solvable with technical informational exchanges and networking, training, and education. One way to build capacity in this MAR aspect of groundwater management is to plan and develop multibenefit projects that provide not only recharge but environmental and recreational benefits, so that many different stakeholders receive something tangible from MAR project investments. Economic cost‐benefit aspects are also solvable; however, elected government officials have to be well‐informed on the complex issues and severity of issues that need to be addressed, along with being involved in capacity building to achieve the political will to take actions.

Decision‐makers in the MCMA could also consider taking a step back with the idea of filling the 800 Mm^3^/year recharge volume needed, and perhaps assess the MAR potential, by starting with available source waters, geographic areas, existing infrastructure for conveyance, recharge area compatibility, and recharge needs in terms of groundwater level declines and subsidence. Mapping the source water (perennial stream flow, rainfall runoff, and recycled water) availability to geographic area and conveyance could help focus future MAR projects to prioritize and size projects based on source water availability. This step could in turn facilitate some financial planning to determine the funding needed to complete the MAR projects needed to stabilize groundwater levels in the MCMA. Once the funding needs are known, planning can commence to determine how to finance MCMA groundwater sustainability in the future. Finally, the disincentives for MAR have to be transformed into incentives, and must also include a water accounting framework and implementation of sound groundwater management practices to be successful and sustainable into the future.
